# Expression of Sympathetic Nervous System Genes in Lamprey Suggests Their Recruitment for Specification of a New Vertebrate Feature

**DOI:** 10.1371/journal.pone.0026543

**Published:** 2011-10-27

**Authors:** Daniela Häming, Marcos Simoes-Costa, Benjamin Uy, Jonathan Valencia, Tatjana Sauka-Spengler, Marianne Bronner-Fraser

**Affiliations:** Division of Biology, California Institute of Technology, Pasadena, California, United States of America; University of Birmingham, United Kingdom

## Abstract

The sea lamprey is a basal, jawless vertebrate that possesses many neural crest derivatives, but lacks jaws and sympathetic ganglia. This raises the possibility that the factors involved in sympathetic neuron differentiation were either a gnathostome innovation or already present in lamprey, but serving different purposes. To distinguish between these possibilities, we isolated lamprey homologues of transcription factors associated with peripheral ganglion formation and examined their deployment in lamprey embryos. We further performed DiI labeling of the neural tube combined with neuronal markers to test if neural crest-derived cells migrate to and differentiate in sites colonized by sympathetic ganglia in jawed vertebrates. Consistent with previous anatomical data in adults, our results in lamprey embryos reveal that neural crest cells fail to migrate ventrally to form sympathetic ganglia, though they do form dorsal root ganglia adjacent to the neural tube. Interestingly, however, paralogs of the battery of transcription factors that mediate sympathetic neuron differentiation (dHand, Ascl1 and Phox2b) are present in the lamprey genome and expressed in various sites in the embryo, but fail to overlap in any ganglionic structures. This raises the intriguing possibility that they may have been recruited during gnathostome evolution to a new function in a neural crest derivative.

## Introduction

Lampreys are agnathans (jawless vertebrates) that have many essential vertebrate characteristics but lack the sympathetic nervous system and jaws. Morphologically, they resemble Cambrian era fossils [1], [2], suggesting a resemblance to the common ancestor of jawless (Agnatha) and jawed (Gnathostomata) vertebrates. Lamprey and hagfish, the only modern agnathans, are likely to be monophyletic, though there remains controversy on this point [3], [4]. As basal vertebrates, both occupy a critical phylogenetic position for understanding emergence of vertebrate traits. However, lamprey offers a significant advantage for developmental studies due of the accessibility and ease of obtaining embryos for experimental manipulation.

Neural crest cells are one of the defining features of vertebrates. This population of multipotent cells gives rise to a variety of different tissues and cell types including cartilage and bone of the facial skeleton, pigment cells, sensory and peripheral ganglia, among other derivatives [5], [4]. The peripheral nervous system of jawed vertebrates is comprised of sensory, parasympathetic, sympathetic and enteric ganglia that form clusters of neurons that innervate peripheral structures and relay information back to the central nervous system. All of these sensory and autonomic ganglia are derived from the neural crest, together with a contribution of cranial placodes to the sensory ganglia of the head [6].

Previous studies have shown that lampreys possess neural crest cells and many neural crest derivatives [7], [8], [9], like cartilage, pigment cells and neurons. In fact, the gene regulatory network underlying the formation and differentiation of neural crest cells is remarkably similar to that of higher vertebrates [10]–[12]. Interestingly, however, lampreys lack some key neural crest structures including dentine, bone and sympathetic neurons.

The sympathetic nervous system is a branch of the autonomic nervous system, responsible for the physiological modulation of inner organs in the absence of conscious control by the central nervous system (CNS). Lampreys and hagfishes lack the chain of sympathetic ganglia chain observed in gnathostomes [13]. Instead, their sympathetic innervation comes from preganglionic fibers that extend directly to the terminal plexus, similar to what is observed in amphioxus [14]. Nevertheless, lamprey and hagfish have been reported to have scattered chromaffin-like cells along blood vessels, the heart and cloaca [15]. Although these cells have been described as analogous to postganglionic neurons [14], it is not yet clear if they connect with the central nervous system and/or represent an evolutionary precursor to the gnathostome sympathetic nervous system [15].

There are a handful of characteristic markers for sympathetic neurons, including Phox2b, Ascl1 (Ash1), and dHand (hand2). Phox2b is a homeodomain transcription factor expressed in several types of neurons in the developing nervous system [16]. The bHLH transcription factor achaete-scute homolog 1 (Ascl1 formerly ash1) is a proneural gene that influences neuronal fate. Ascl1 is expressed in some domains of the neuroepithelium of the forebrain and in precursors of sympathetic and enteric neurons [17]. dHand is a basic helix-loop-helix transcription factor that is essential for proliferation and noradrenergic differentiation of sympathetic neuron precursors during development [18]. Here, we asked whether this suite of genes exists in lamprey and if so, where they were expressed.

To address this question, we isolated lamprey homologues of these genes and examined their expression patterns in embryos by *in situ* hybridization at various stages of development. The results show that all three genes are individually found in different areas of the head with only Phox2 expressed in cells at the trunk level. DiI labeling of presumptive neural crest cells failed to show a neural crest contribution to sites where sympathetic ganglia would be expected to coalesce. In contrast, DiI labeled neural crest cells contributed to both dorsal root ganglia and enteric ganglion cells of the gut. Taken together, the results raise the intriguing possibility that the transcriptional program responsible for migration to and/or differentiation within the site of sympathetic ganglion formation in gnathostomes was assembled through the recruitment of Phox2b, dHand and Ascl1 by precursors derived from the neural crest.

## Results

While it has been established that lamprey lacks an organized sympathetic nervous system [14], [19], some studies suggest that scattered sympathetic neurons and chromaffin cells might be associated with blood vessels, hindgut, cloaca and kidneys [15]. Furthermore, it has been suggested that lamprey has sympathetic innervation to the heart [13], [20], as opposed to spinal adrenergic innervation as seen in other vertebrates. To investigate the organization of the sympathetic nervous system in lamprey embryos, we examined neural crest migration in the vagal and trunk regions, and mapped the expression pattern of markers characteristic of differentiating sympathetic neurons.

### DiI and neurofilament labeling of trunk neural crest in lamprey

In the trunk region of gnathostomes, neural crest precursors to sensory and sympathetic ganglia migrate from the dorsal neural tube, along a ventral pathway to coalesce either next to the neural tube, to form dorsal root ganglia, or further ventrally adjacent to the dorsal aorta, to form sympathetic ganglia.

**Figure 1:**
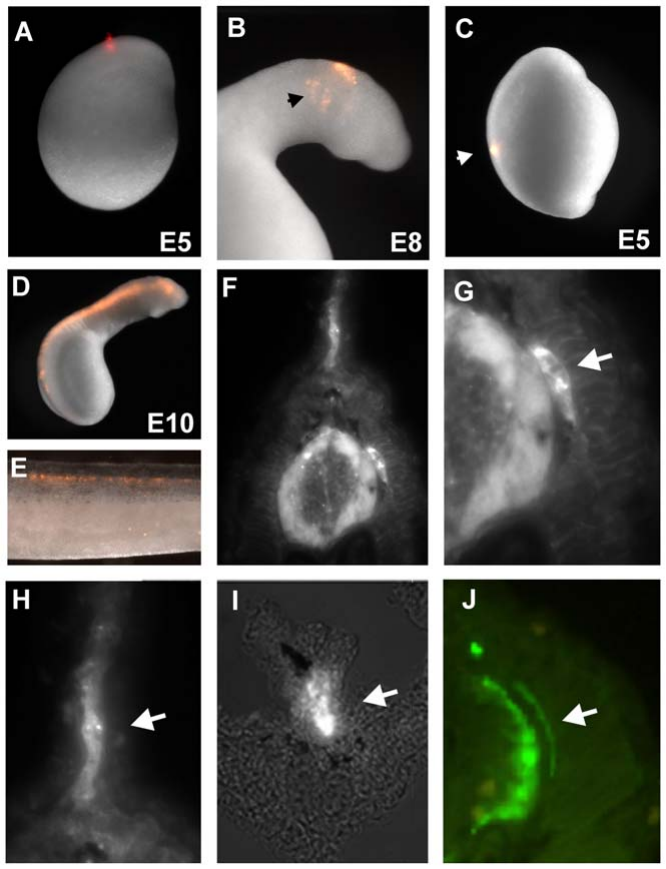
DiI labeling of lamprey neural crest cells reveals absence of sympathetic ganglia during embryonic development. (A) Focal injections of DiI in the lamprey neural tube at day 5 results in labeling of migrating cephalic neural crest (arrow in B). However, focal injections into the posterior neural tube (C) fail to label trunk neural crest cells. D) Filling the lumen of the neural tube with DiI after cavitation produces labeled trunk neural crest cells in several neural crest derivatives (E). A section through an injected embryo (F) shows labeling of the dorsal root ganglia (DRG, arrow in G), the mesenchyme of the fin (H) and neurons surrounding the gut (F), but no structure that resembles sympathetic ganglia. Neurofilament staining (J) labels neural crest derivatives such as the DRG but also fails to reveal any sympathetic like structures.

To test whether lamprey neural crest cells migrate ventrally to contribute to sensory and/or sympathetic ganglia as in gnathostomes, we first performed focal injections of the lipophilic dye, DiI into the dorsal neural tube at trunk levels. In lamprey, the neural tube forms by secondary neurulation, where a solid rod-like structure transforms into a tube whose lumen forms by cavitation. At embryonic day 5, the neural rod elevates, gradually detaching from the dorsal epithelium. The first indication of neural crest precursors occurs at this time. The head morphologically extends and becomes visible at day 6, concomitant with cavitation. Neural crest primordia at this stage appear as bulges on the dorsal aspect of the newly formed neural tube [21]. Focal injections of DiI performed at any level of the trunk dorsal neural rod prior to cavitation (day 5.5 to day 6) (Figure 1A) failed to give rise to any migrating neural crest. In contrast, similar injections into the head neural folds at similar stages resulted in labeling of the migrating crest (Figure 1A and 1B).

Accordingly, we performed DiI-labeling at later times, after cavitation at day 6.5 to day 7, by injecting dye into the lumen of the neural tube (Figure 1D). This approach resulted in labeling of migrating neural crest cells. In embryos receiving neural tube injections at day 6.5 to 7 and examined through day 34, the labeled cells contributed to several neural crest derivatives at trunk and vagal levels (Figure 1E). These include dorsal root ganglia (DRGs) (Figure 1F and 1G), the mesenchymal cells of the fin (Figure 1H), as well as enteric ganglia (Figure 1I). However, no structures resembling sympathetic ganglia were observed at any stage. These results suggest that lamprey neural crest cells contribute to dorsal root ganglia but fail to condense into sympathetic-like structures during embryonic development.

To examine neuronal differentiation, we performed immunostaining using antibodies against neuronal markers. At day 16, anti-neurofilament staining was observed in the neural tube, dorsal root ganglia (Figure 1J) and also in the ventral part of the gut, likely staining enteric ganglia. Some embryos were allowed to develop until day 31 at which time the dorsal root ganglia continued to express neurofilament protein and appeared somewhat larger in size than at earlier stages (data not shown). However, at no time point did we note neurofilament staining in the vicinity of the dorsal aorta where sympathetic ganglia coalesce in gnathostomes.

### Expression pattern of transcription factors associated with sympathetic neuron differentiation in the lamprey

In gnathostomes, several transcription factors have been implicated in sympathetic nervous system formation. These include the basic-helix-loop helix factors, dHand and Ascl1, as well as the homeodomain transcription factor Phox2b. To examine the presence and deployment of these lamprey genes during neural crest development, we cloned fragments of lamprey Hand, Phox2 and Ascl1 orthologues using 5′RACE and determined their expression patterns by *in situ* hybridization.

**Figure 2:**
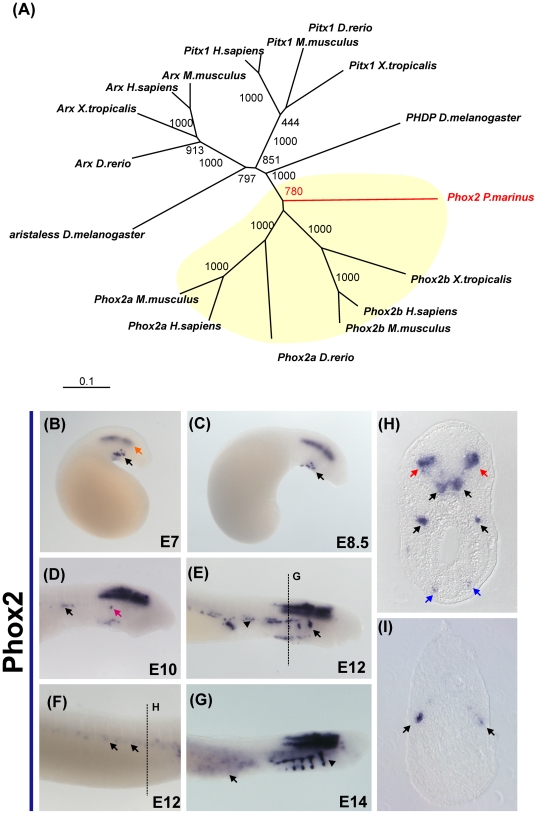
Expression of the transcription factor Phox2 in embryos of *P. marinus*. (A) Phylogenetic analysis of a putative Phox2 fragment places it at the base of the gnathostome Phox2a and Phox2b gene families. (B) Expression of Phox2 is initially observed on the hindbrain (red arrow) and on a group of cells of the ventral mesenchyme (black arrow). (C) This expression pattern is maintained at day 8.5, as Phox2 positive cells migrate ventrally (black arrow). (D) At day 10, a stream of positive cells is observed above the heart and appears to be migrating posteriorly (black arrow). At this stage, Phox2 transcripts are first detected on the epibranchial ganglia (red arrow). (E) At day 21, the expression domain of Phox2 expands to include cranial (black arrow) and epibranchial ganglia (arrowhead). A section of this embryo (H) reveals staining in the motor neurons of the hindbrain, epibranchial ganglia and ventral mesenchyme (red, black and blue arrows, respectively). (F) At posterior axial levels, Phox2b expressing cells are observed adjacent to the yolk sac (section on I). (G) At later stages, a larger number of cells around the yolk start expressing Phox2 (black arrow), and there is strong expression in cranial nerves (arrowhead).

Phylogenetic analysis places the putative Phox2 lamprey ortholog at the base of the branch containing gnathostome Phox2a and Phox2b genes (Figure 2A). This suggests the duplication event that gave rise to these paralogs took place after cyclostomes diverged from the vertebrate lineage. Expression of lamprey Phox2 was first observed in hindbrain motor neurons and the ventral branchial mesenchyme at day 7 and 8 (Figure 2B and C). By day 10, there was additional expression in the epibranchial ganglia and cranial nerves (Figure 2D). Expression of Phox2 was also observed in individual cells in a medial stream reminiscent of cells migrating posteriorly (Figure 2D). On day 12, expression was maintained in the hindbrain, cranial nerves and ventral mesenchyme (Figure 2E and 2H). Moreover, the expression domain expanded to include the anterior epibranchial ganglia (arrow on Figure 2E). In the trunk, the medial expression first observed on day 10 appeared to expand posteriorly (Figure 2F and I). At later stages, Phox2 transcripts also were detected in cells surrounding the yolk (E14, arrow on Figure 2G) and in the notochord.

**Figure 3:**
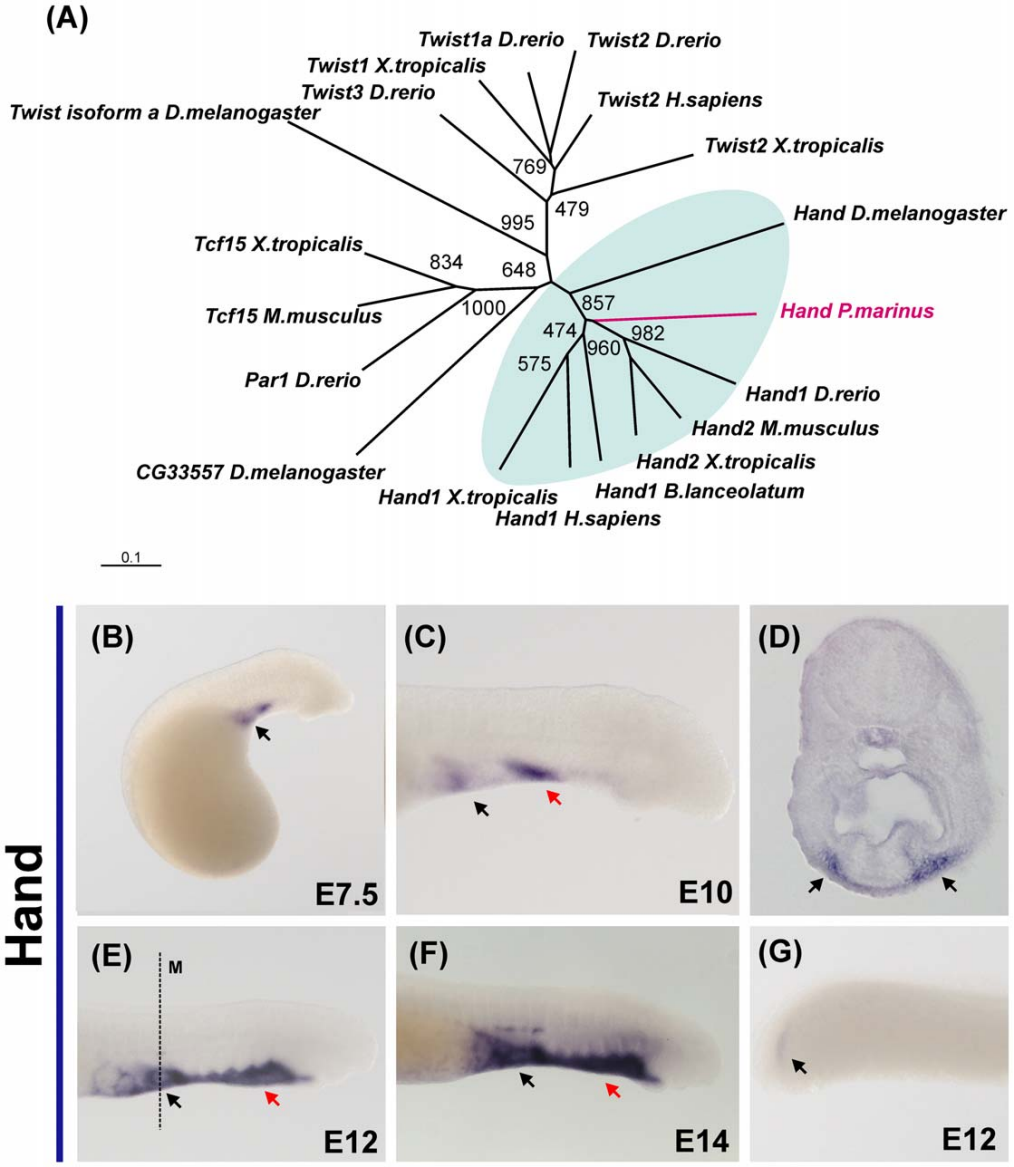
Expression of the helix-loop-helix transcription factor Hand in lamprey embryos. (A) Phylogenetic analysis suggests lamprey has one ortholog of both the dHand and eHand gnathostome genes. (B) Lamprey Hand expression is first observed in the cardiac field, and is conspicuous after 7 days of development. (B) At day 10, two domains of expression are clearly present: the heart (black arrow) and a part of the anterior mesenchyme (red arrow). (C) Expression in the cardiac ganglia (black arrow) is first detected at day 12 days; transverse sections reveal high abundance of transcript in the mesenchyme flanking the pharynx (black arrows on F). (E) Strong expression is observed in the ventral mesenchyme of the lamprey head (read arrow), as well as the heart at day 14 (black arrow). (G) Posterior expression appears to be restricted to the unsegmented mesoderm of the tail (black arrow).

The Hand ortholog isolated from lamprey clusters to the base of the branch that contains both the D-Hand and E-hand gnathostome genes in our phylogenetic analysis (Figure 3A). Lamprey Hand is first observed at day 5 in the bilateral precursors that form the cardiac field (data not shown). From day 7 to day 10, there is additional staining visible in the anterior portion of the ventral mesenchyme (Figure 3D and 3F). At day 12, in addition to the heart, the entire ventral mesenchyme that surrounds the endostyle and the notochord expresses Hand (Figure 3D and 3F). Hand transcripts were also detected at day 14 in the cardiac ganglia (Figure 3D and 3E) and in the posterior mesoderm (Figure 3G).

**Figure 4:**
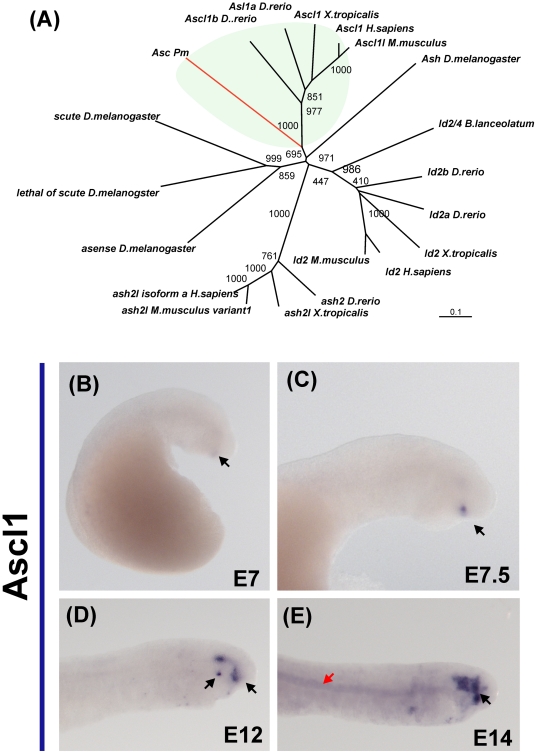
Expression of Ascl1 during embryonic development of the lamprey. (A) Phylogenetic analysis places the putative lamprey Ascl1 ortholog with gnathostome Ascl1 genes. (B) Onset of Ascl1 expression is apparent at day 7, when transcripts are detected in the anterior lip mesoderm. Shortly afterwards (C), faint expression is observed in the lens (arrow). (D) On day 12, lens expression is elevated (arrow), and staining on the hypophysis and trigeminal ganglia (red arrow) are also observed. (E) Finally, on day 14, Ascl1 expression is observed in the notochord (red arrow).

The lamprey Ascl1 fragment was within the branch that contains the Ascl1 gnathostome orthologues (Figure 4A). Expression of Ascl1 was first noted at day 6, with very faint expression observed in the pituitary gland (Figure 1B). This pattern was maintained at day 7, accompanied by additional expression in the lens (Figure 4C) By day 10, Ascl1 was upregulated in the anterior lip mesoderm (day 10) and by day 12, transcripts also were detected in the VI cranial ganglion (Figure 4D). From day 11 onward, Ascl1 also was expressed faintly in the notochord (Figure 4G).

In addition, analysis of the expression of these three transcription factors in lamprey juveniles through *in situ* hybridization performed in tissue slices failed to reveal any co-expression in ganglionic structures (data not shown). Thus, we were unable to identify a structure resembling sympathetic ganglia in the lamprey embryo in which Hand, Ascl1 and Phox2 were co-expressed. Our results, in concert with the neurofilament and DiI data, demonstrate that the lamprey does not possess a sympathetic nervous system analogous to that in gnathostomes. We were also unable to identify any cells that might represent precursors to sympathetic postganglionic neurons of cyclostomes.

## Discussion

We analyzed the expression patterns of three known marker genes of the gnathostome sympathetic nervous system for their deployment in lamprey embryos. At 9 and 11 days after fertilization, we found expression of Hand, Ascl1 and Phox2 in the head region, with only Phox2 showing additional expression in a stream of cells that migrate toward the trunk. These cells may correspond to lateral line cells derived from ectodermal placodes. In ganglia, we see only Hand expressed in the cardiac ganglia and Phox2 in the epibranchial ganglia.

Interestingly, there was no overlapping expression of these “sympathetic” genes in any domain in the embryo. Consistent with this marker analysis, we found no contribution of DiI-labeled neural crest cells to structures adjacent to the dorsal aorta, where sympathetic ganglia arise in gnathostomes, despite finding robust labeling of other neural crest derivatives like dorsal root ganglia and enteric neurons. In addition, the absence of neuronal staining with a neurofilament antibody supports the lack of sympathetic ganglia, while revealing the presence of dorsal root and enteric ganglia. Taken together, our results suggest that the lamprey does not have a definitive sympathetic nervous system.

In gnathostomes, Ascl1 is generally required for development of autonomic neurons, with expression initiating earlier than Phox2b. Phox2b also is required for autonomic neurogenesis and, in a feedback loop, is required for maintenance of Ascl1 expression [22]. Ascl1 induces expression of pan-neuronal genes in neural crest precursor cells of the peripheral nervous system, but does not specify subtype specific expression of tyrosine hydroxylase (TH) or dopamine-β-hydroxylase (DBH), the enzymes responsible for the catalyzing synthesis of the neurotransmitter, nor-epinephrine [23]. Loss-of-function of dHand, another determinant of the sympathetic lineage, blocks neural crest cell differentiation into noradrenergic neurons, whereas its over-expression upregulates Phox2b, TH and DBH. Expression of dHand depends on Phox2b, but not Ascl1 [22].

Classical literature regarding the lamprey sympathetic nervous system is scarce and contradictory. Lampreys possess several neurotransmitters including acetylcholine and noradrenaline in the central nervous system. In addition, sequenced fragments from lamprey DNA reveal the presence of two adrenergic receptors [24] that, in gnathostomes, are the most abundant in the sympathetic nervous system. While this suggests the presence of sympathetic activity in cyclostomes, ganglionic structures are absent in the trunk and tail of the lamprey. Only a sympathetic ganglion formed by small intensely fluorescent cells (SIF cells) has been described adjacent to the adult heart [25], [26]. It is possible that such a ganglion would be innervated by the vagus nerve, which is cardio-inhibitory in all vertebrates, with the exception of the cyclostomes [27]. Additionally, it has been suggested that cardiovascular function is controlled by chromaffin cells, located on the wall of blood vessels, kidney and urogenital ducts [28].

SIF cells and chromaffin cells are closely related, and SIF cells are considered to be the intermediate in morphology between chromaffin cells and sympathetic neurons [28]. One intriguing possibility is that there may have been a shift from chromaffin and SIF cells to sympathetic neurons during gnathostome evolution, such that chromaffin and SIF cells represent the evolutionary precursor to sympathetic neurons. These three cell types are closely related lineage-wise, sharing a sympathoadrenal progenitor which co-expresses markers characteristic of both chromaffin cells and sympathetic neurons [28], [29].

Our DiI labeling experiments show a discrete neural crest stream that migrates towards the heart (the cardiac neural crest; data not shown) which could be the source of the progenitors that give rise to the cardiac SIF cell aggregation [26]. Indeed, there is expression of Hand in ganglia adjacent to the heart (figure 3F), although Phox2 is not present in the same structures. However, at the stages examined, we failed to observe migratory precursors that might give rise to the scattered chromaffin cells that are said to occur throughout the lamprey body.

In conclusion, our results indicate that no structures homologous to sympathetic ganglia arise during lamprey embryogenesis. While lamprey may possess a blueprint for the sympathetic nervous system comprised of chromaffin and SIF cells, a more detailed analysis of the molecular identity of such cells would be necessary to establish them as true phylogenetic precursors of sympathetic neurons. Thus, our data suggests that sympathetic neurons are a gnathostome innovation, and that the recruitment of Phox2, Hand and Ascl1 into a new gene battery allowed for the emergence of this new neural crest derivative.

## Materials and Methods

### DiI labeling in lamprey embryos

5 –7 day old embryos were dechorionated in 0.1xMMR and placed into agarose-coated petridishes. DiI solution (0.5 µg/µl, prepared in 0.3M sucrose) was filled into glass needles and injected into distinct neural crest population or into the entire neural tube. Embryos were analyzed for the injection into a discrete location or into the neural tube by fluorescence microscopy. Embryos were raised in petri dishes containing 0.1×MMR at 18°C and the migration of DiI stained cells was analyzed every day. Once the embryos had reached the desired stage they were fixed using 4%Paraformaldehyde in PBS at room temperature for 1hr.

### Obtention of lamprey orthologs through 5′ Rapid Amplification of cDNA ends (RLM-5′ RACE)

Orthologs of Phox2b, dHand and Asc1 were identified by bioinformatic survey of the lamprey genomic sequences and cloned using RACE. Total RNA was extracted from embryos using the Ambion: RNAquous kit. RLM-5′ RACE was conducted on the total mRNA in accordance with Invitrogen: GeneRacer Kit. Total RNA was dephosphorylated through Calf Intestinal Phosphatase (CIP) treatment, decapped via Tobacco Acid Pyrophosphatase (TAP), ligated with the GeneRacer RNA oligo, and finally reverse transcribed using random hexamer priming to form the cDNA template. The genes of interest were amplified using touch down PCR and cloned with TOPO TA Cloning Kit (Invitrogen).

### Immunostaining of lamprey embryos

Immunostaining of lamprey embryos was performed as previously described [30]. Neurofilament (NF-M) antibody was used 1∶200 in blocking solution. As a secondary antibody Alexa 488 anti mouse IgG2a was used 1∶1000 in blocking solution. Sections were degelatinized in 42°C PBS for 10 minutes and washed with PBSTr 2 times for 5 minutes. Afterwards the sections were blocked in 10% goat serum in PBSTr at 4°C for 5 hrs. The blocked sections were incubated overnight at 4°C with the neurofilament (NF-M) antibody 1∶200 in blocking solution. To remove unbound antibody the sections were washed 5 times for 10 minutes with PBSTr. As a secondary antibody Alexa 488 anti mouse IgG2a 1∶1000 in blocking solution was used for 2 hrs at room temperature. Unbound secondary antibody was washed of 3 times for 10 minutes in PBSTr followed by 2 washes for 10 minutes in PBS. For mounting, sections were dipped into distilled water a few times and mounted with Permaflour.

### Whole-mount in situ hybridization on lamprey embryos

Whole-mount lamprey *in-situ* was performed as previously described [21]. Plasmid DNA templates used for digoxigenin-labeled RNA probes were Hand, Ascl1 and Phox2.

Embryos fixed in MEMFA for 1 hr at room temperature. For detection of transcripts, embryos were bleaches with a 10% hydrogen peroxide solution for 10 minutes, washed with PBSTw (DEPC) 3 times for 5 minutes, and were treated with 20 µg/ml proteinase K in PBSTw (DEPC) for 10 minutes. Following this incubation the proteinase K solution was replaced with 2 mg/ml glycine in PBSTw (DEPC) for 10 minutes. After this treatment the embryos were postfixed with 4% PFA for 20 minutes. Afterwards the embryos were pre-hybridized for 3 hrs in hybridization solution at 70°C, and incubated in hybridization solution containing 1–10 µg/ml labeled RNA probe for 16 hrs at 70°C. To remove unbound probe the embryos were washed twice for 15 minutes with hybridization solution at 70°C and then 4 times for 45 minutes. Afterwards the embryos were washed with hybridization solution and MABT 1∶1 at 70°C for 30 minutes followed by four washes in MABT only at room temperature for 30 minutes wash. Blocking of the embryos was done for 4 hrs in blocking solution. The embryos were subsequently incubated with the Anti-DIG-AP antibody 1∶2000 in blocking solution over night at 4°C. To remove unbound antibody, embryos were washed with MABT for 2 times 5 minutes, 2 times 30 minutes, 6 times 1 hr and an overnight wash at 4°C. For the color reaction the embryos were adjusted to NTMT buffer by 4 washes for 15 minutes. For the color reaction we used BM purple. The embryos were incubated in BM purple, covert from light, until the desired staining intensity was reached. To stop the color reaction the embryos were washed in PBSTw 3 times for 5 minutes and fixed again in 4% paraformaldehyde for 2 hrs at room temperature.

### Embedding and sectioning of lamprey embryos

Lamprey embryos were washed with PBSTr 3 times 15 min. Subsequently they were incubated in 15% sucrose in PBS for 3 hrs at room temperature, 7.5% gelatin and 15% sucrose for 12 at 37°C, and 20% gelatin for 4 hrs at 37°C. Subsequently embryos were positioned in 20% gelatin and frozen with liquid nitrogen. Embryos were sectioned at 8 – 10 µm with a Microm HM550 cryostat.

### Phylogenetic Analysis

Alignments were built with the coding sequences retrieved from GenBank. Neighbor Joining (NJ) tree were constructed using ClustalX protocol from the DNA STAR package. The trees were visualized using Tree View v. 0.5.0.
